# Effect of the Protic vs. Non-Protic Molecular Environment on the *cis* to *trans* Conformation Change of Phototrexate Drug

**DOI:** 10.3390/ijms252312703

**Published:** 2024-11-26

**Authors:** Flórián Bencze, László Kiss, Heng Li, Hui Yan, László Kollár, Sándor Kunsági-Máté

**Affiliations:** 1 Institute of Organic and Medicinal Chemistry, Faculty of Pharmacy, University of Pécs, Honvéd útja 1, H-7624 Pécs, Hungary; benczeflori.123@gmail.com (F.B.); kissl@gamma.ttk.pte.hu (L.K.); 2 Department of Physical Chemistry and Materials Science, Faculty of Sciences, University of Pécs, Ifjúság útja 6, H-7624 Pécs, Hungary; 3 János Szentágothai Research Center, Ifjúság útja 20, H-7624 Pécs, Hungary; kollar@gamma.ttk.pte.hu; 4 Fujian Provincial Key Laboratory of Semiconductors and Applications, Collaborative Innovation Center for Optoelectronic Semiconductors and Efficient Devices, Department of Physics, Xiamen University, Xiamen 361005, China; liheng3000@xmu.edu.cn; 5 Jiujiang Research Institute, Xiamen University, Jiujiang 332000, China; 6 Tianjin Key Laboratory of Photoelectric Materials and Devices, School of Materials Science and Engineering, Tianjin University of Technology, Tianjin 300384, China; yanhui@tjut.edu.cn; 7 Key Laboratory of Display Materials and Photoelectric Devices, Tianjin University of Technology, Ministry of Education, Tianjin 300384, China; 8 HUN-REN-PTE Research Group for Selective Chemical Syntheses, Ifjúság u. 6., H-7624 Pécs, Hungary

**Keywords:** phototrexate, photopharmacology, photoswitchable drug, isomer stability

## Abstract

The therapeutical applicability of the anticancer drug phototrexate, a photoswitchable derivative of the antimetabolite dihydrofolate reductase inhibitor methotrexate, highly depends on the stability of its bioactive isomer. Considering that only the *cis* configuration of phototrexate is bioactive, in this work, the effect of the molecular environment on the stability of the *cis* isomer of this drug has been investigated. UV-vis absorption and fluorescence-based solvent relaxation methods have been used. Protic methanol and non-protic dimethylsulfoxide were used as medium-ranged permittivity solvents. The results showed a decreased rate of *cis → trans* conversion and enhanced stabilities of the *cis* isomer in methanol. Temperature-dependent measurements of the isomerization rate reflect the increased activation energy in methanol.

## 1. Introduction

Due to the off-target toxicity, low therapeutic indices, and nonspecific targeting of most chemotherapeutics, the need to fix these problems has garnered interest over the last decades [1,2]. To overcome the related difficulties, one of the recent research fields is photopharmacology. The principle of photopharmacology involves introducing a photoswitchable unit into the molecular structure of a bioactive compound [3].

Synthetic photoswitches can be suitable tools to activate the drugs exclusively at their target site. This procedure is a promising approach to improve cancer therapies. Photoswitches themself are chromophores that can be reversibly isomerized when exposed to light. The field of research that attempts to control biological activity with these molecules is called photopharmacology [4].

Azobenzene is the most widely used photoswitch in biological applications because of its ease of synthesis and functionalization, fast photo-isomerization, and the low rate of photo-bleaching [5,6]. Phototrexate (PHX) is a photoswitchable azobenzene analogue of methotrexate [7] that has been synthesized and described by C. Matera et al. [8] and by Mashita et al. [9]. PHX contains a diazene stereogenic unit, and its pharmacological activity is higher in its *cis* state than in the more thermodynamically stable *trans* state. The transition between the two isomers is reversible. No hysteresis was observed while repeating the switching several times.

**Figure 1:**
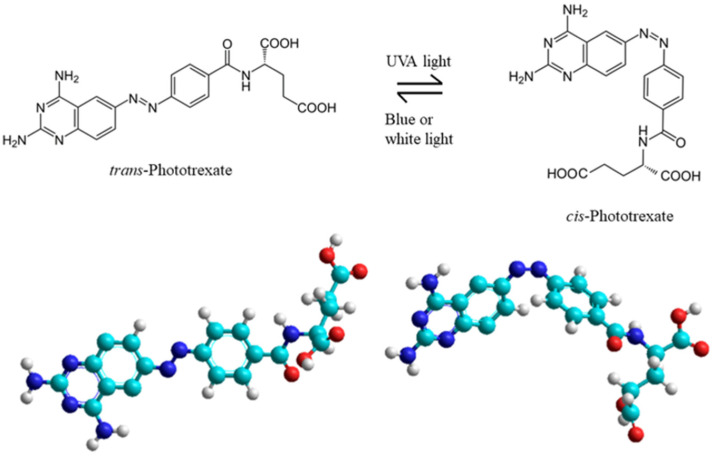
Chemical structures of *trans*-Phototrexate (*trans*-PHX) and *cis*-Phototrexate (*cis*-PHX) and the reversible isomerization of PHX. Bottom: 3D structure of the two isomers of the PHX: *trans*-PHX (left) and *cis*-PHX (right).

PHX can be effectively isomerized from *trans* to *cis* with UVA light (375 nm) and backisomerized from *cis* to *trans* with blue (460 nm) or white light (Figure 1). The antifolate effect only appears in light-exposed regions and decreases in dark regions. The target tissues exposed to UV-illumination are primarily the skin, the digestive, respiratory, and reproductive tracts. We mention here that the wavelength of light applied to induce the *trans* to *cis* conversion can be even longer by substituting all four *ortho* positions with methoxy groups in an amidoazobenzene derivative; this makes *trans*-to-*cis* photoswitching possible using green light (530 to 560 nm) [10]. The conversion also can be affected by adsorption to gold nanoparticles [11]. Other photoswitches are also known, such as combretastatin A-4 analog, in which the photoswitch is based on replacing the azobenzene structure with a stilbene bridge [12].

To support the therapeutic application of the photoswitchable phototrexate, the formation of inclusion complexes of PHX with two cavitand derivatives in dimethyl sulfoxide medium has been investigated in our previous studies [13]. Photoluminescence methods have been applied to determine the complex stabilities and the related enthalpy and entropy changes associated with the complex formation at around room temperature. We found that the formation of inclusion complexes of PHX with cavitands is a promising method to regulate the pharmacokinetics of such drug molecules.

However, according to the effectiveness of the therapy, the stability of the *cis* configuration must be high enough such that the drug can take effect. From another point of view, it is necessary for the drug to be converted back to the biologically inactive *trans* isomer after the effect has taken place, and thus, only the *trans* form of the drug should leave the body.

For these reasons, this work examines how changes within the molecular environment can affect the stability of the bioactive *cis* form of this drug. In this case, the effect of a protic vs. non-protic molecular environment on the *cis*-PHX stability will be compared.

## 2. Results and Discussion

### 2.1. Effect of the Composition of the Solvent Mixture on the Absorption Spectra of PHX

**Figure 2:**
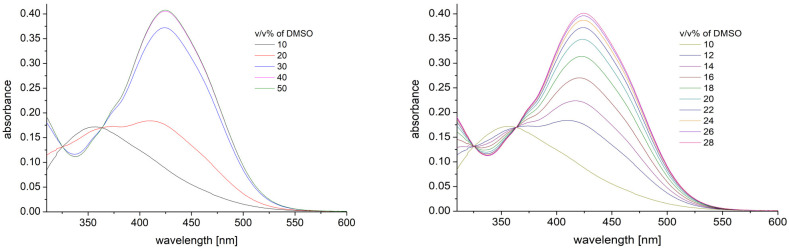
Changes of absorption spectra of PHX with 50 μM concentrations in differently composed methanol–DMSO mixtures. (**Left**) DMSO concentration varies within the 10 vol % and 50 vol % range. The spectra remain unchanged above 50 vol % DMSO. (**Right**) DMSO concentration varies within the range of 10 vol % and 28 vol %.

The absorption spectra of 50 μM phototrexate in mixtures of methanol and DMSO were recorded. Due to the decreased solubility of PHX in methanol, the composition of the solvent mixtures were varied within the 10 vol % and 100 vol % DMSO concentrations. In DMSO, a strong peak at 425 nm is detected, and this peak decreases with the methanol content of the solution (Figure 2 Left). In contrast, a peak at 350 nm appears at a high methanol concentration, while the peak measured in pure DMSO solvent disappeared. However, these significant spectral changes happened within a narrow range of the solvent composition, around 15–20 vol % of DMSO content (Figure 2 Right). This agrees with our previous observation in mixtures of primary alcohols [14] or water and ethanol [15].

**Figure 3:**
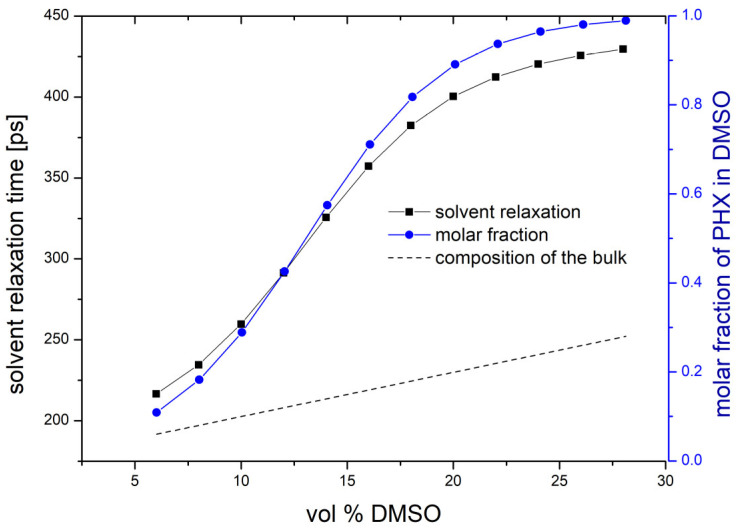
Correlation of the solvent relaxation times and the composition of the solvation shell of phototrexate molecules estimated from the absorption spectra. DMSO concentration varies within the range of 6 vol % and 28 vol %.

The considerable spectral changes obtained with solutions of low DMSO concentrations are probably related to the different compositions of the solvation shell of PHX molecules. Assuming linearly proportional changes of the spectra with the DMSO content of the solvation shell, the spectra reflect much higher DMSO concentration in the solvation shell compared to the DMSO concentration in the bulk (Figure 3). The dashed line indicates the generally expected case when the composition of the bulk and the solvation shell would be the same. According to our previous works (e.g., [16]), this property is associated with the entropy-driven exchange process of differently sized solvent molecules when their binding forces to the solute are the same or at least close to each other. In this particular case, the DMSO molecules are a little larger than the methanol molecules, and the entropy gain associated with the methanol-to-DMSO exchange process can drive the DMSO-rich composition of the solvation shell of the PHX molecules. This description is supported by the strong correlation of the solvent relaxation times with the composition of the solvation shell estimated from the absorption spectra. The increased solvent relaxation times reflect the slower motion of the larger DMSO molecules within the solvation shell of the PHX.

The property of the solvent relaxation times being the lowest in the methanol-rich environment reflects the presence of the individual methanol molecules instead of methanol clusters in the solvation shell of PHX (e.g., [17,18]). This property also suggests that the interaction between the methanol and PHX molecules is stronger than the interaction between the methanol molecules themselves.

### 2.2. cis to trans Formation Kinetics of PHX Molecules in Solvent Mixtures

The thermodynamically more stable and pharmacologically inactive *trans* isomer of PHX was isomerized to the *cis* configuration using UV light (λ = 366 nm) for 15 min while the samples were stirred. In both samples, when PHX was dissolved in pure DMSO or in a solvent containing mainly methanol, the absorption spectra of the mixture reflected the PHX reaching the equilibrium of isomerization within 10 min. We mention here that ^1^H NMR measurements reflect the non-perfect isomerization of PHX under these experimental conditions.

It is worth mentioning that the *trans*-PHX → *cis*-PHX isomerization can be tracked by ^1^H NMR. The pairs of doublets at 8.10 ppm (8.4 Hz), 7.93 ppm (8.4 Hz) and 7.84 ppm (8.3 Hz), 6.98 ppm (8.3 Hz) are perfectly separated in the case of *trans*-PHX and *cis*-PHX, respectively. Therefore, the integrals of the 2,6- and 3,5-protons of the *para*-substituted aryl ring can be used for quantitative analysis. According to the ^1^H NMR analysis carried out in pure DMSO-*d*_6_, 30%, 39% and 42% of the starting *trans*-PHX was converted to the *cis* isomer in 5, 10 and 20 min irradiation time. The same conversions can be obtained by using the integral of the more complex signal of the α-proton (C*H*(COOH)) in the glutamate moiety at 4.44 ppm and 4.36 ppm (*trans*-PHX and *cis*-PHX, respectively).

**Figure 4:**
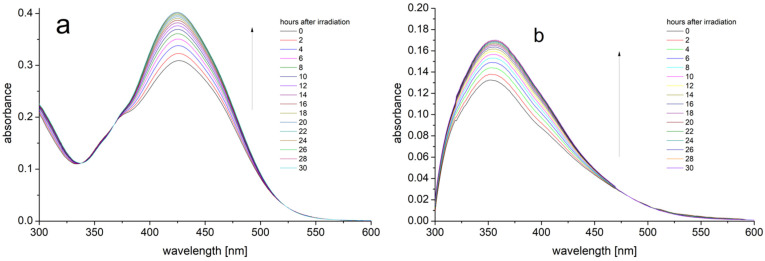
Shows the time-dependent representative absorption spectra of PHX in two cases: (**a**) the solvent composed of pure DMSO and (**b**) the solvent composed of 90 vol % methanol and 10 vol % DMSO.

After the isomerization, the absorption spectra were recorded at 2 h intervals for 30 h. Figure 4 shows the time-dependent representative absorption spectra of PHX in two cases: (a) the solvent composed of pure DMSO and (b) the solvent composed of 90 vol % methanol and 10 vol % DMSO.

**Figure 5:**
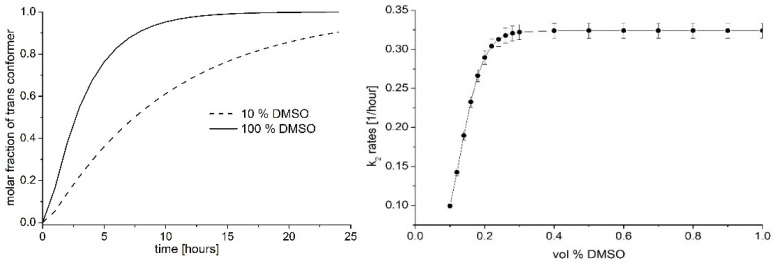
(**Left**) Representative *cis* → *trans* conversion curves of PHX in two cases: the solvent is pure DMSO or consists of 90 vol % methanol and 10 vol % DMSO. (**Right**): k_2_ plotted against vol % DMSO.

Figure 5 Left shows the representative *cis* → *trans* conversion curves of PHX in two cases: the solvent composed of pure DMSO or the solvent consisting of 90 vol % methanol and 10 vol % DMSO. The curves are fitted to the normalized spectra of the *trans* isomer (considered as the perfect conversion). In contrast, the spectra were recorded just after irradiation (100% of molecules are in *cis* conformation, considered as 0% conversion). Since the shape of the absorption spectra remained unchanged during the *cis* → *trans* conversion, the maximum values were plotted against the time. The shape of the curves suggests consecutive conversion reactions, where the first step is very fast (k_1_ = 2.012 1/h), while the k_2_ rates are varied between 0.977 1/h and 0.325 1/h while the DMSO concentration increases from 10 vol % up to 100 vol %.

Figure 5 Right shows the dependence of the k_2_ reaction rate as a function of the composition of the binary solvent. The figure clearly shows that the rates increase very quickly with the DMSO content of the solutions, which correlates to the changes in both the solvent relaxation times and the DMSO content of the solvation shell calculated from the spectral data.

### 2.3. Activation Energies Associated with the cis-to-trans Conversion of PHX in Different Media

**Figure 6:**
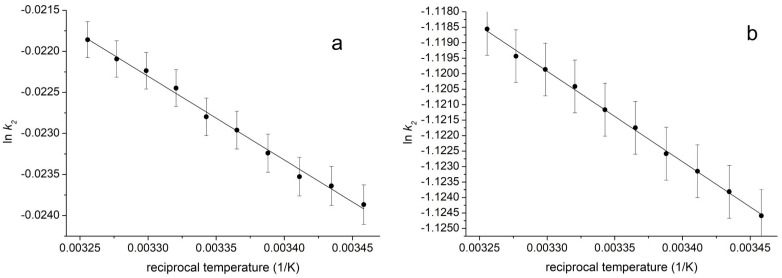
Arrhenian plots of the rates of the second conversion step associated with the *cis* → *trans* conversion of PHX in two cases: (**a**) the solvent composed of pure DMSO and (**b**) the solvent composed of 90 vol % methanol and 10 vol % DMSO. The slopes determine 86 kJ/mol and 234 kJ/mol activation energy for the (**a**,**b**) processes, respectively.

The activation energies associated with the *cis* → *trans* isomerization of PHX have been investigated and compared in two representative cases: the solvent of pure DMSO or the solvent consisting of 90 vol % methanol and 10 vol % DMSO. Spectrofluorimetric measurements were performed at 10 different temperatures within the 289.16 K to 307.16 K range in both cases. PL intensities were measured at 490 nm; after that, the intensity values of the excitation at 366 nm were evaluated. The time-dependence of the PL intensities followed the time-dependence of the absorption spectra exactly, so in the same way, Equation (1) was fitted to the normalized intensity values (see Figure 5 Left), and then the ln*k_2_* values were plotted against the reciprocal temperature. Figure 6 shows the related Arrhenian plots.

The slopes of the curves in Figure 6a,b reflect that the activation energy of the *cis* → *trans* conversion of PHX increases from 86 kJ/mol to 234 kJ/mol when the solvent is changed from pure DMSO to methanol-rich 10 vol % DMSO and 90 vol % methanol solvent mixture. The increased activation energy could probably only be obtained at a very low (<15 vol %) DMSO concentration since the solvent relaxation and the reaction rates show similar dependence on the solvent composition. Unfortunately, we are unable to confirm this idea. This is because determining the activation energies assumes temperature-dependent measurements of the reaction rates. Still, the temperature will undoubtedly also affect the formation thermodynamics of the solvation shells. Therefore, the composition of the solvation shells would also change, resulting in a non-linear dependence of ln *k* values on the reciprocal temperatures.

### 2.4. A Model to Describe the Possible Reason of Elevated Activation Energies in Methanol

**Figure 7:**
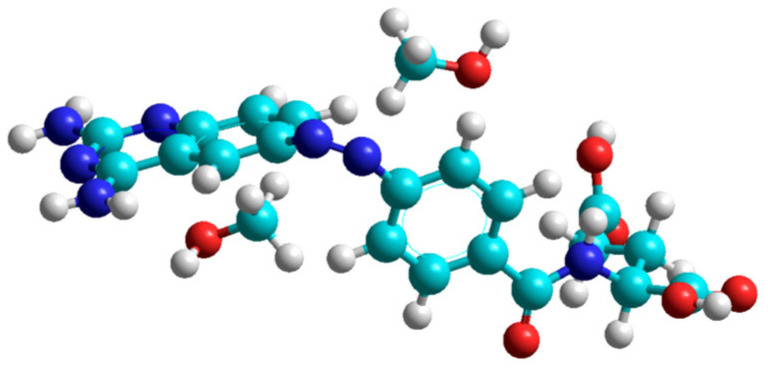
Optimized structure of the coordination of two methanol molecules to the *cis*-PHX. (HF/6-31G* level of calculation) The methanol molecules are stabilized preferably by the hydrogen bonds.

We have focused on the azobenzene unit of the molecular skeleton and optimized the coordination of solvent molecules close to the azobenzene bridge. The results support the coordination of two methanol molecules to the bridge of *cis*-PHX, while no coordination was observed in the case of *trans*-PHX due to the steric hindrance of the nearest aromatic hydrogen atoms. Similarly, no coordination of the DMSO molecules has been observed in both cases of *cis*- and *trans*-PHX molecules. This phenomenon is probably due to the steric hindrance of the coordination of the larger DMSO molecule to the azobenzene unit. Given these results, we can conclude that the elevated activation energy of *cis* → *trans* conversion of PHX in methanol is probably a consequence of the elimination of methanol molecules from the diazo bridge during the transformation process. However, the interaction energy of two methanol molecules with the PHX molecule have been calculated as 76 kJ/mol (Figure 7), which is much lower than the difference of the activation energies determined experimentally (234 − 86 = 148 kJ/mol). Unfortunately, considering the limitation of the quantum chemical model used here, the theoretical and experimental values cannot be directly compared. Anyway, these results highlight the necessity of further investigations to clarify other aspects relevant to the enhanced stability of *cis*-PHX in methanol.

## 3. Materials and Methods

### 3.1. Synthesis of PHX

**Figure 8:**
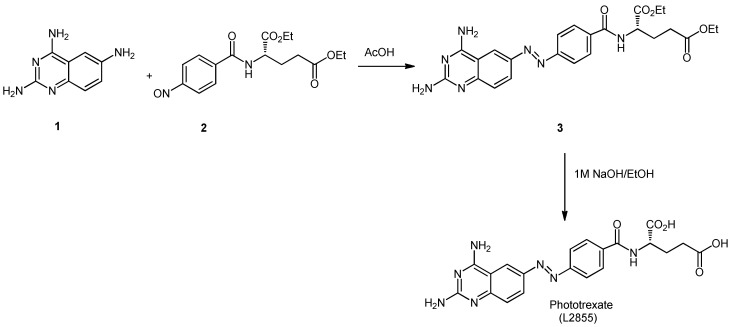
Schematic scheme of the synthesis of PHX.

*Trans*-Phototrexate (*trans*-PHX) was synthesized in our institute following the procedure of Matera et al. [8]., published in more detail in [19]. Briefly explained, **1** quinazoline-2,4,6-triamine was conjugated to **2** (S)-diethyl 2-(4-nitrosobenzamido)-pentanoate to offer compound **3**. The latter was hydrolyzed in a mixture of sodium hydroxide and ethanol to yield PHX (Figure 8).

After chromatographic purification, the physicochemical data of PHX agreed with the earlier published data. HRMS (ESI): *m*/*z* [M + H]^+^ calculated for C_20_H_20_N_7_O_5_^+^: 438.1520; found: 438.1520 (Figure 9 in [13]). IR: 3325, 2780, 2696, 1568, 1411, 1363, 1043, 1009, 924, 839, 766, 645, 618, 496 cm^−1^.

### 3.2. Other Chemicals and Instruments

The applied solvents, dimethyl sulfoxide (DMSO) and methanol (spectroscopic grade), were purchased from Merck (Darmstadt, Germany).

### 3.3. Instrumentation

UV-vis spectra were recorded by a Specord Plus 210 spectrophotometer (Analytik Jena, Jena, Germany).

Fluorometric measurements, including the solvent relaxation measurements, were performed with a Fluorolog τ3 spectrofluorometer (Jobin-Yvon/SPEX, Longjumeau, France). Fluorescence spectra were recorded using either a 350 nm or 425 nm excitation wavelength in methanol or DMSO-rich solutions, respectively. The shift of the emission band was used for data evaluation. For data collection, a photon counting method with a 0.1 s integration time was used, 2 nm bandwidths were set, and quartz cuvettes with 1.0 cm thickness were applied.

The thermodynamically stable but pharmacologically inactive *trans*-PHX was isomerized by applying UV light (λ = 366 nm) provided by a Fluotest lamp (Original Hanau, Hanau, Germany). Each sample was irradiated for 20 min within a quartz cuvette used later in the measurement. The samples were tempered during measurement using the tempered sample holder of the Specord Plus 210 spectrophotometer. A Grant thermostat (Grant LTD6G, Grant Instruments, London, UK) was used for controlling temperature. To minimize the irradiation during photometric measurements, a 500 nm/minute scanning rate with a 1 nm bandwidth was applied. IR spectra were obtained with a Bruker Alpha FT-IR instrument (Bruker Optics, Ettlingen, Germany) with ATR support on a diamond plate.

### 3.4. Sample Preparation

10^−3^ M stock solution of PHX was prepared in DMSO. This stock solution was then diluted by appropriate amounts of DMSO and methanol to prepare the samples where the concentration of the PHX was kept constant at 50 μM, while the DMSO concentration of the samples was varied within the 5 vol % and 100 vol % range.

### 3.5. Modelling

Quantum chemical calculations to study the coordination of methanol and DMSO molecules to *cis* and *trans* PHX were performed using HyperChem 8.0 (HyperCube Inc.; Gainesville, FL, USA) code [20]. The semiempirical PM3 method (e.g., [21]), followed by ab initio HF calculations using the 6-31G* basis set (e.g., [22]), were chosen according to previous studies on similar molecular systems and due to their simplicity, achieving the results in an appropriate amount of CPU time.

### 3.6. Data Evaluation

The detailed mechanism of the *cis* → *trans* conversion of PHX is unknown, but the spectral changes vs. time curve reflect a two-step consecutive reaction where the first step is very fast and the slow second step determines the overall reaction rate. Accordingly, the following equation was fitted to the spectral data:

(1) $$c_{c}=c_{0}\left[1-\frac{1}{\left(k_{2}-k_{1}\right)}(k_{2}e^{-k_{1}t}-k_{1}e^{-k_{2}t})\right],$$

where *C_c_* is the concentration of the *trans*-PHX, *C*_0_ is the initial concentration of the *cis*-PHX, and *k*_1_ and *k*_2_ reflect the rate constants of the different conversion steps.

## 4. Conclusions

In this study, the stability of the biologically active configuration of the anticancer drug phototrexate was investigated according to its therapeutical applicability. The bioactive *cis* isomer of PHX shows a higher stability in a protic methanol environment, while the presence of about 15 vol % DMSO drastically increases the conversion to the thermodynamically more stable *trans* form of PHX. Solvent relaxation measurements reflect the faster motion of the methanol molecules within the solvation shell of PHX than the DMSO molecules. This property suggests the presence of individual methanol molecules instead of larger methanol clusters within the solvation shell. Further investigations are planned to clarify this observation.

## Data Availability

The data presented in this study are available upon request from the corresponding author. The data are not publicly available due to institutional regulation.
